# The Influence of Dentine on the pH of Calcium Hydroxide, Chlorhexidine Gel, and Experimental Bioactive Glass-Based Root Canal Medicament

**DOI:** 10.1155/2015/686259

**Published:** 2015-08-04

**Authors:** Ceci Nunes Carvalho, Laila Gonzales Freire, Alexandre Pinheiro Lima de Carvalho, Evandro Luiz Siqueira, José Bauer, Giovana Cunha Gritti, Juliana Pereira de Souza, Giulio Gavini

**Affiliations:** ^1^University Ceuma, Rua Josué Montello No. 1, Renascença II, 65075-120 São Luís, MA, Brazil; ^2^School of Dentistry, University of São Paulo, Department of Restorative Dentistry, Endodontics Division, Avenida Prof. Lineu Prestes 2227, 05508-000 São Paulo, SP, Brazil; ^3^Department of Dentistry I, School of Dentistry, University Federal of Maranhão (UFMA), Avenida dos Portugueses, s/n, 65085-680 São Luís, MA, Brazil; ^4^Center of Materials Science and Technology, Energy and Nuclear Research Institute (IPEN), Avenida Lineu Prestes 2242, Cidade Universitaria, 05588-900 São Paulo, SP, Brazil

## Abstract

*Objectives*. To evaluate the influence of dentine on the pH of different medications in standardized simulated canals.* Materials and Methods*. Forty resin blocks were divided into groups with and without dentine powder, as follows: 2% chlorhexidine gel; 2% chlorhexidine gel associated with calcium hydroxide PA; calcium hydroxide PA delivered in propylene glycol 600; and NPG delivered in distilled water. The dentine powder was obtained from the root dentine of bovine teeth and added to the medications. The simulated canals were placed in containers with 1.5 mL of deionized water and pH was monitored in multiple intervals, up to 30 days. The mean pH values were calculated and submitted to statistical analysis using paired Student's *t*-test and ANOVA complemented by the Tukey test (*p* < 0.05). *Results*. There was no statistical difference between the groups with and without dentine powder (*p* > 0.05). The pH values of calcium hydroxide were significantly higher than those of NPG in the first 24 hours (*p* < 0.05). After 7 days, both behaved in a similar manner.* Conclusion*. The addition of dentine powder to the medications evaluated did not alter the pH of the external solution in any of the time points tested.

## 1. Introduction

The difficulty in eliminating microorganisms that remain in the root canal system even after cleaning and shaping procedures demonstrates the need to complement chemomechanical preparation by using an intracanal medication with antimicrobial properties.

Calcium hydroxide has clinical indications well-documented in the literature [1]. Its mechanism of action is directly attributed to its ability for dissociation into calcium and hydroxyl ions resulting in an increase in the local pH, which alters the biological properties of bacteria, leading to bacterial cell toxicity [2].

However, calcium hydroxide is not equally effective against all microorganisms found in the root canal system [3]. It has been reported that* Enterococcus faecalis* shows a resistance to elevated pH, an ability to penetrate dentinal tubules, and an ability to adapt to different environmental conditions [4]. For this reason, new antimicrobial agents and associations have been introduced, in an effort to find a better alternative to calcium hydroxide.

Chlorhexidine has a wide spectrum of antibacterial and antifungal actions, enhanced by pH levels between 5.5 and 7.0 [5, 6]. Its association with calcium hydroxide has been recommended in an attempt to amplify its antimicrobial effect [7].

More recently,* in vitro* studies indicate that bioactive glasses can provide an alternative to calcium hydroxide in intracanal interappointment medication, since their action spectrum and antimicrobial efficacy are similar [8].

Because these biomaterials are bioactive, they can induce dentinal remineralization [9] and release ions when in contact with the dentinal fluid, thus promoting an alkaline pH in the environment [10]. Furthermore, it is known that the dentinal structure has a buffer effect on wide pH variations and may be responsible for reducing the antimicrobial action of medications within the root canal [11]. In contrast, it has been reported that the antibacterial efficacy of bioactive glass increases when mixed with dentine [12].

Therefore, the objective of this study was to evaluate the influence of dentine on the pH of calcium hydroxide, 2% chlorhexidine gel, and bioactive niobium phosphate glass in standardized simulated canals. The experimental hypothesis tested was that dentine influences the pH of medications based on calcium hydroxide, chlorhexidine gel, and bioactive niobium phosphate glass.

## 2. Methods

### 2.1. Dentine Powder

Ten bovine incisors were used to obtain the dentine powder. They were cleaned with ultrasound and stored in physiological saline until the start of the experiment. All the teeth were sectioned perpendicularly to their long axis, with the aid of diamond disks (Buehler, Lake Bluff, IL, USA), just below the cementoenamel junction. All intraroot dentine was removed with long-neck #4 spherical carbide burs (KG-Sorensen, São Paulo, SP, Brazil) at low speed and without refrigeration and immediately stored in plastic vials [13].

### 2.2. Preparation of the Bioactive Niobium Phosphate Glass

Phosphate glass was prepared by melting mixtures of diammonium phosphate (Reagent Grade—Casa Americana, São Paulo, SP, Brazil), niobium oxide (Optical Grade—Companhia Brasileira de Mineração e Metalurgia, Araxá, MG, Brazil), calcium oxide (Reagent Grade—Casa Americana) and sodium carbonate (Reagent Grade—Casa Americana) in an electric furnace. Afterwards, the chemical compounds were mixed in a shaker-mixer for 1 hour, placed in an alumina crucible, and heated in an electric furnace (Lindberg Blue M, Benton Harbor, MI, USA). The material was then heated to 1400°C for complete melting of the precursors, and the liquid was poured into a stainless steel mold and cooled to room temperature. The glass was then crushed in a vibrating system with a tungsten ball (Pulverisette, Fritsch, Germany) for 30 minutes [14].

### 2.3. Preparation of the Medicaments

Forty resin blocks with simulated canals and a standardized apical foramen measuring 400 *μ*m in diameter were used [15].

The canals were divided into groups according to the medications tested: 2% chlorhexidine gel (Fórmula & Ação, São Paulo, SP, Brazil); 2% chlorhexidine gel with dentine powder added; 2% chlorhexidine gel associated with calcium hydroxide PA (Fórmula & Ação); 2% chlorhexidine gel associated with calcium hydroxide PA, with dentine powder added; calcium hydroxide PA delivered in propylene glycol 600; calcium hydroxide PA delivered in propylene glycol, with dentine powder added; bioactive niobium phosphate glass delivered in distilled water; and bioactive niobium phosphate glass delivered in distilled water, with dentine powder added.

The medications were prepared using a glass slab and a spatula, with the aid of a precision scale, using a ratio of 1 g of powder to 1 mL of liquid. In the groups with dentine powder, the powder was added to the proportion of 1.8% of the volume of medication [16].

Subsequently, the pastes were inserted into the simulated canals through a plastic syringe with a needle. Complete filling of the canals was verified by observing medication overflow through the apical foramen and backflow through the canal orifice. Next, the access cavities were sealed with a resin composite (Z350; 3M ESPE, St. Paul, USA) and cyanoacrylate (SuperBonder Instant Adhesive; Loctite Corp., Cleveland, OH, USA).

### 2.4. pH Measurement

The five simulated canals of each group were placed separately in containers with 1.5 mL of deionized water. After 10 minutes, 24 hours, and 7, 14, 21, and 30 days, the canals were placed in fresh containers with deionized water, and the medium pH changes were measured with a pH meter (pH meter, Model E520, Metrohm, Herisau, Switzerland) with a microelectrode sensitive to hydrogen ions.

The microelectrode was calibrated at pH 7 and pH 4 with standardized solutions before each measurement, was washed thoroughly with deionized water after use, and was dried with absorbent paper in order to eliminate any residue.

The pH of the deionized water in the containers was measured before immersion of each specimen.

Thus, five measurements for each group/period were made and the mean pH values were calculated and submitted to statistical analysis using paired Student's *t*-test to analyze the influence of dentine, and ANOVA was complemented by the Tukey test (*p* < 0.05) to analyze the groups at the different experimental time points.

## 3. Results

Table 1 shows the mean pH values of the medications for the different time periods studied. No significant statistical differences were found between the groups with and without dentine powder (*p* > 0.05).

The pH values of calcium hydroxide, alone or associated with chlorhexidine, were significantly higher than those of bioactive niobium phosphate glass up to the first 24 hours (*p* < 0.05). After 7 days, both behaved in a similar manner.

## 4. Discussion

The present study showed that the addition of dentine powder to the medications evaluated did not alter the pH of the external solution in any of the time periods tested. Freire et al. found the same result for the pH of calcium hydroxide delivered in propylene glycol; however, the pH of 2% chlorhexidine gel, whether associated with calcium hydroxide or not, was increased significantly by the presence of dentine powder [13]. Along the same line, a study evaluated the influence of dentine on the pH of medications and found that the results for dentine obtained from the root canal walls were different from those for dentine obtained from the pulp chamber floor. The addition of dentine from canal walls significantly reduced the pH of calcium hydroxide after 14 days [17].

Most interactions between intracanal medications and dentine appear to have a negative impact on their performance. Haapasalo et al. [11] demonstrated that dentine powder has an inhibiting effect on medications for intracanal use and that the increase in pH promoted by calcium hydroxide can be checked by the buffering capacity of dentine. Initially, a high concentration of dentine (18% w/v) was used in the inhibition experiments [11]. However, another study [18] showed that even 1.8% dentine (w/v) totally prevented the killing of* E. faecalis* by a saturated calcium hydroxide solution.

The study model, using standardized simulated canals, was chosen to standardize the amount of medication used in each sample [15, 17].

Because of the inhibition of antimicrobial agents commonly used in endodontics by the presence of dentine, inhibition of the antimicrobial activity of bioactive glasses was expected [19]. However, contrary to expectations, preliminary experiments demonstrated an additive antimicrobial effect of BAG S53P4 in the presence of dentine [12].

Bioactive glass is a material that is activated when in contact with tissue fluids, inducing an alkaline pH, similar to medications based on calcium hydroxide [8]. Although the antimicrobial effect of bioactive glasses is not completely understood, it may be related to a pH increase in aqueous suspensions [20] and also be potentiated in the presence of dentine [12].

Bioactive niobium phosphate glass behaved similarly to calcium hydroxide after 7 days; however, in the first 24 hours, the latter reached significantly higher pH values than those of the former. One possible explanation for this is that the chemical stability of phosphate glasses increases when niobium is added [21, 22], thus leading to a slightly slower release of ions. The dissolution of vitreous materials depends significantly on the pH of the solution. The leaching rate of phosphate glasses increases significantly in acidic environments. This occurs because the dissolution of glass occurs predominantly by the process of hydration and not hydrolysis [23]. In acid mediums, chains of phosphates are protonated, with rupture of the ionic cross-link connections between the chains. In basic mediums, the leaching process of phosphate glasses also occurs, albeit at lower rates compared to acid mediums and at higher rates compared to neutral mediums [22, 23]. This fact may render the use of phosphate glass as an intracanal medication an interesting option, considering that the essential purpose of intracanal medication is to promote decontamination of the root canal system in necrotic pulp cases, most of which present an acidic environment. Dentine powder could also act as a receptor for the ions in solution and, therefore, act as a catalyst in the dissolution of the glass in aqueous suspension, interfering with bacterial viability [24]. The antimicrobial activity of bioactive glass against* E. faecalis* has been considered moderate when compared to 2% chlorhexidine gel, 2% metronidazole gel, and calcium hydroxide [10].

It is known that the pH elevation effect produced by calcium hydroxide causes an alteration in the integrity of the bacterial cytoplasmic membrane, leading to cellular destruction [1]. The results of the present study showed that the pH of calcium hydroxide remained slightly alkaline during the 30 days of the study. Freire et al. [13] found that the pH of calcium hydroxide remained elevated whether or not dentine powder was present, with values ranging from 12.5 to 14.0 during the 21 days of the study, whereas, in a similar study, [17] observed values above 12 during 14 days.

It should be highlighted that calcium hydroxide pastes produce an extremely high level of alkaline pH when placed directly in solution. This same effect is not observed when they are placed within the root canal, requiring that the medication diffuses throughout the entire dentine [16]. Hence, the solubility of calcium hydroxide is lower and saturation is attained at a relatively low concentration of hydroxyl ions [11].

To avoid a cumulative effect, the water in which the blocks were immersed was replaced after each experimental period [15, 16]. This procedure may explain the maintenance of the pH value, from the 7-day time point until the end of the experiment, for all samples, even those with low volumes of deionized water.

Chlorhexidine has been used in association with calcium hydroxide in an attempt to increase the antimicrobial effects of this substance owing to the wide spectrum of actions and substantivity of chlorhexidine [7]. When these medications are associated, elevated pH levels may cause precipitation of chlorhexidine, since its optimal action pH is between 5 and 7 [25, 26]. In the present study, this association produced a mean pH above 7, as also observed in other studies [5, 27, 28]. Hence, the usefulness of associating calcium hydroxide to chlorhexidine remains controversial. Agrafioti et al. [17] showed that the pH of 2% chlorhexidine gel was significantly altered by the presence of dentine, raising it to values much higher than optimal for inducing antimicrobial activity. These results concur with those of some previous reports [13, 19].

Other studies should be conducted to evaluate the performance and antimicrobial activity of bioactive niobium phosphate glass before its clinical use as an intracanal medicament can be proposed.

## Figures and Tables

**Table 1:** Mean pH values of the medications for the six time periods of the study.

Medication	Immediate	24 hours	Days				*p* value
			7	14	21	30	
CHX	6.72^a^	5.52^a^	6.78^a^	6.36^a^	5.7^a^	5.90^a^	0.3530
CHX + D	6.78^a^	5.40^a^	6.54^a^	6.56^a^	6.44^a^	6.12^a^	

Ca(OH)_2_ + CHX	9.02^b^	7.66^c^	7.44^b^	7.24^b^	7.34^b^	6.88^b^	0.0550
Ca(OH)_2_ + CHX + D	9.06^b^	8.22^c^	7.40^b^	7.48^b^	7.60^b^	7.58^b^	

Ca(OH)_2_ + PEG	10.24^c^	8.40^c^	7.38^b^	7.42^b^	7.52^b^	7.68^b^	0.5530
Ca(OH)_2_ + PEG + D	11.00^c^	8.14^c^	7.40^b^	7.48^b^	7.50^b^	7.66^b^	

BNPG + distilled water	7.42^a^	7.22^b^	7.56^b^	7.30^b^	7.90^b^	7.30^b^	0.9202
BNPG + distilled water + D	8.32^a^	7.12^b^	7.22^b^	7.20^b^	7.20^b^	7.50^b^	

Different superscript letters in rows indicate significant differences between time periods (*p* < 0.05).

CHX: chlorhexidine gel; Ca(OH)_2_: calcium hydroxide; PEG: propylene glycol 600; BNPG: bioactive niobium phosphate glass; and D: dentine.
